# The prevalence and associated factors of overweight/obesity and abdominal obesity in South-eastern of Iran: a cross-sectional study based on Rafsanjan cohort study

**DOI:** 10.1186/s12889-023-15700-0

**Published:** 2023-05-11

**Authors:** Tabandeh Sadeghi, Narjes Soltani, Zahra Jamali, Fatemeh Ayoobi, Parvin Khalili, Ali Shamsizadeh, Mostafa Nasirzadeh, Ali Esmaeili‑Nadimi, Carlo La Vecchia, Zahra Jalali

**Affiliations:** 1 Department of Pediatric Nursing, School of Nursing and Midwifery, Non-Communicable Diseases Research Center, Rafsanjan University of Medical Sciences, Rafsanjan, Iran; 2 Non-Communicable Diseases Research Center, Rafsanjan University of Medical Sciences, Rafsanjan, Iran; 3 Department of Epidemiology, School of Public Health, Social Determinants of Health Research Center, Rafsanjan University of Medical Sciences, Rafsanjan, Iran; 4 Physiology-Pharmacology Research Center, Rafsanjan University of Medical Sciences, Rafsanjan, Iran; 5 Department of Health Promotion and Education, School of Health, Occupational Environment Research Center, Rafsanjan University of Medical Sciences, Rafsanjan, Iran; 6 Department of Clinical Sciences and Community Health, University Degli Study Di Milano, Milan, 20133 Italy; 7 Department of Clinical Biochemistry, School of Medicine, Non-Communicable Diseases Research Center, Rafsanjan University of Medical Sciences, Rafsanjan, Iran; 8 Occupational Safety and Health Research Center, NICICO, World Safety Organization and Rafsanjan University of Medical Sciences, Rafsanjan, Iran

**Keywords:** Obesity, Overweight, Abdominal obesity, Cohort study, Rafsanjan Cohort Study, Prospective Epidemiological Research Studies in Iran (PERSIAN)

## Abstract

**Background:**

Obesity has become a major health issue in both high and middle-income countries, increasing the risk of cardiovascular diseases and all-cause mortality. Risk of obesity is related to both unchangeable factors such as genetics and gender, and modifiable lifestyle factors. Most importantly, finding the major modifiable lifestyle factors which contribute to obesity may provide valuable benefits to every society. This study aimed to determine the association of demographic and lifestyle parameters with overweight/obesity and abdominal obesity in a population of Iranian adults.

**Methods:**

In this cross-sectional study, adult participants of Rafsanjan Cohort Study (RCS) (as one of the district areas of the PERSIAN cohort (Prospective Epidemiological Research Studies in IrAN) included the study population. RCS is a population-based prospective cohort of men and women aged 35–70 years, launched in August 2015. Individuals were recruited from four urban and suburban areas of Rafsanjan, south-eastern of Iran. Trained experts interviewed each participant and completed the related questionnaires about his/her socioeconomic status, demography, anthropometric features, personal habits, physical activity and medical history. Multinomial logistic regression models were used to examine the relationships between overweight/obesity/abdominal obesity and associated factors.

**Results:**

From 9980 participants, 1974 (42.42%) males and 2115 (39.70%) females were overweight, 784 (16.85%) males, 2223 (41.73%) females were obese and 1895 (40.73%) males and 989 (18.57%) females were normal weight. Also, 832 (17.9%) males and 4548 (85.4%) females had abdominal obesity and 3819 (82.1%) males and 778 (14.6%) females didn’t have abdominal obesity. Based on the adjusted multiple logistic regression, overweight/obesity (BMI > 25) was associated with age > 45, female gender, education ≥ 13 years, heavy physical activity, wealth status index (WSI), alcohol consumption, current cigarette smoking and opium consumption compared to reference group. Also, odds of abdominal obesity displayed a significant association with age > 45, female gender, education > 5 years, physical activity, WSI, current cigarette smoking, alcohol and opium consumption compared to reference group.

**Conclusions:**

Our results recommend local public health strategies that promote training the society on the health benefits of avoiding alcohol, getting more physical exercise and gaining more personal education on the health-threatening lifestyle.

**Supplementary Information:**

The online version contains supplementary material available at 10.1186/s12889-023-15700-0.

## Introduction

Obesity is one of the most prevalent public health problems in both high and middle-income countries [1]. Lifestyle changes toward an inactive life or eating more, are the major cause of obesity epidemic [2].

In adults, the most common criteria to evaluate the obesity is body mass index [BMI][3]. BMI ≥ 30 kg/m2 is defined as obesity and also, BMI 25–29.9 kg/m2 as overweight [4]. Obesity especially abdominal obesity, is associated with cardiovascular diseases and all-cause mortality [5–7]. According to the third National Health and Nutrition Examination Survey study, normal-weight central obesity (high waist-to-hip ratio) displayed higher association with cardiovascular mortality than obesity defined by BMI [7].

According to the World Health Organization, in 2016, more than 1.9 billion and 650 million adults were overweight and obese respectively, this is responsible for 2.8 million deaths annually [8]. In Iran, the prevalence of obesity in women and men were different in various regions, although all of them indicated a high prevalence of overweight and obesity [9, 10].

Obesity is a determinant risk factor for diabetes, cardiovascular disease, cancer, sleep problems and osteoarthritis. It can affect the quality of human life in various aspects including physical, social, economic and psychological conditions [8, 11–13]. In the United States, a positive association was observed between obesity and depression. The results showed that physical and social dysfunction as well as mental overeating are able to play mediating roles for this association [14]. The obesity epidemic is developing in low and middle-income countries, especially in urban areas [8, 11].

One of the most important strategies of the World Health Organization to control the obesity is to increase the physical activity and healthy diet [1]. Therefore, in designation of helpful strategies, a comprehensive understanding of the current situation and health determinants is important [15]. Studies which provide data available for evaluation of central and abdominal obesity and their risk factors in Rafsanjan city located in the southwest of Iran are scarce [4]. The aim of the present study was to investigate the current epidemic of overweight/obesity, abdominal obesity and their related factors in the adult population of the Rafsanjan cohort study (RCS)[16].

## Methods

The data for this study were collected in Rafsanjan cohort study (RCS) [16] as a part of the Prospective Epidemiological Research Studies in Iran (PERSIAN) [17]. RCS was designed to enroll 10,000 participants of both genders aged 35–70 years, including both urban and suburban areas. In each cluster, 2500 participants (35–70 year) were enrolled (10,000 participants).

### Variables assessment

Trained experts interviewed each participant and completed the related questionnaires about his/her socioeconomic status, demography, personal habits, physical activity, and medical history. The anthropometric characteristics were measured by trained health professionals at RCS Center.

All questionnaires were previously validated in the PERSIAN cohort study [17]. Marital status was categorized into married and single including never married, divorced, widowed or other. The daily physical activity of the participants was weighted based on its relative metabolic cost, known as a metabolic equivalent (MET), and MET-h/day for 24 h is derived in this way. Then MET was classified into three categories including low ( < = 35.299), moderate (35.3-40.325) and heavy ( > = 40.326) groups respectively based on the 25th and 75th percentiles. Data on the lifestyle habits including cigarette smoking was coded as never, current and former smokers, Alcohol drinking and opium consumption were coded as yes (currently or formerly) and no (never).

Socio-economic status was measured according to the Wealth score index (WSI). The wealth score index (WSI) was classified into four categories based on 25th, 50th and 90th percentiles: low income (1st group: ≤ − 0.6069), low-middle income (2nd group: − 0.607 to 0.0349), middle-high income (3rd group: 0.035 to 1.169) and high income (4th group: ≥ 1.170).

BMI was calculated from height and weight data as kg/m2. Overweight was defined as BMI 25–29.9 kg/m2 and obesity as BMI ≥ 30 kg/m2. Waist circumference ≥ 102 cm for men and ≥ 88 cm for women was defined as abdominal obesity based on NCEP-ATP III criteria [18]. The frequency difference between total number and some of the covariates was related to missing data.

### Statistical analysis

Stata software V.12 was used for all the statistical analyses. Means and standard deviations (SD) were calculated for continuous variables, and proportions were calculated for the categorical variables. Between-group comparisons were made by independent t-test and chi-square test. Multinomial logistic regression models were used to examine the relationships between overweight/obesity/abdominal obesity and associated factors by adjusting for covariates. Potential confounding variables were sequentially entered into model according to their hypothesized strengths of association with overweight/obesity/abdominal obesity. Variables with a p-value < 0.25 were selected as confounders. A cut off value of 0.25 is supported by some literature [19, 20]. The adjusted model is adjusted for confounding variables age (continuous variable), gender (male, female), education years (categorical variable), wealth status index (categorical variable), and the lifestyle variables cigarette smoking (categorical variable), alcohol drinking (yes, no) and opium consumption (yes, no), physical activity level (categorical variable) and marital status (categorical variable). All p-values were two-sided, and a significance level of 0.05 was used for p-value.

## Results

According to the results, from 9980 participants, 4089 (40.97%) were overweight, 3007 (30.13%) were obese and 2884 (28.90%) had normal weight. The prevalence of normal BMI, overweight and obesity were shown based on demographic characteristics of individuals in both genders in Table 1. From 4653 male participants, in categorize of BMI, 1974 (42.42%) were overweight, and 784 (16.85%) were obese and from 5327 female participants, 2115 (39.70%) were overweight and 2223 (41.73%) were obese.

**Table 1:** Prevalence of demographic, selected medical and laboratory characteristics of study participants by BMI categorize

Characteristics	BMI in Male							BMI in Female						
	< 25 (n = 1895)	%(CI)	25-29.9 (n = 1974)	%(CI)	≥ 30 (n = 784)	%(CI)	P-Value	< 25 (n = 989)	%(CI)	25-29.9 (n = 2115)	%(CI)	≥ 30 (n = 2223)	%(CI)	P-Value
**Age- yr. no (%)**							0.003							< 0.001
35–45	665	38.2(36.9–40.6)	751	43.2(40.9–45.5)	323	18.6(16.8–20.5)		481	24.3(22.4–26.2)	810	40.8(38.7–43)	692	34.9(32.8–37)	
46–55	552	40.1(37.6–42.8)	585	42.6(39.9–45.2)	238	17.3(15.4–19.4)		260	15.3(13.7–17.1)	678	40(37.7–42.4)	757	44.7(42.3–47)	
≥ 56	678	44(41.6–46.6)	638	41.5(39-43.9)	223	14.5(12.8–16.3)		248	15.1(13.4–16.8)	627	38(35.7–40.4)	774	46.9(44.5–49.4)	
**Marital status- no (%)**							0.019							0.022
Single	52	48.2(38.8–57.7)	37	34.2(25.8–43.8)	19	17.6(11.4–26.1)		151	22.4(19.4–25.7)	251	37.2(33.7–40.9)	272	40.4(36.7–44.1)	
Married	1844	40.6(39.1–42)	1937	42.6(41.2–44)	765	16.8(15.8–17.9)		838	18(16.9–19.1)	1864	40.1(38.6–41.5)	1951	41.9(40.5–43.4)	
**Education-no (%)**							< 0.001							< 0.001
≤ 5 years	546	47.6(44.8–50.5)	433	37.8(35-40.6)	167	14.6(12.6–16.7)		204	17.1(15.6–18.7)	874	37.2(35.3–39.2)	1072	45.7(43.6–47.7)	
6–12 years	1008	39.8(37.9–41.7)	1087	43(41-44.9)	436	17.2(15.8–18.7)		407	17.6(16.1–19.2)	940	40.7(38.7–42.7)	964	41.7(39.7–43.7)	
≥ 13 years	339	34.8(31.9–37.9)	454	46.6(43.5–49.8)	181	18.6(16.3–21.2)		180	27.1(23.8–30.6)	301	45.2(41.5–49.1)	184	27.7(24.4–31.2)	
**Physical activity-no (%)**							< 0.001							< 0.001
Low	563	38.2(35.7–40.7)	650	44(41.5–46.6)	263	17.8(15.9–19.9)		171	15.8(13.8–18.2)	371	34.4(31.6–37.3)	537	49.8(46.8–52.8)	
Moderate	626	37.6(35.30–40)	744	44.7(42.3–47.1)	295	17.7(16-19.6)		587	18(16.7–19.3)	1350	41.3(39.6–43)	1331	40.7(39.1–42.4)	
Heavy	707	46.7(44.2–49.3)	580	38.3(35.9–40.8)	226	15(13.2–16.8)		231	23.6(21-26.3)	394	40.2(37.2–43.3)	355	36.2(33.3–39.3)	
**WSI-no (%)**							< 0.001							0.001
Low	491	54.6(51.3–57.8)	292	32.4(29.5–35.6)	117	13(10.9–15.4)		300	20.8(18.8–23)	553	38.3(35.8–40.9)	590	40.9(38.4–43.4)	
Low-middle	545	41.3(38.7–44)	549	41.6(39-44.3)	226	17.1(15.2–19.3)		268	17.4(15.6–19.3)	595	38.6(36.2–41.1)	680	44(41.6–46.6)	
Middle-high	718	37(34.9–39.2)	895	46.1(43.9–48.3)	328	16.9(15.3–18.6)		355	17.3(15.7–19)	839	40.9(38.8–43.1)	857	41.8(39.7–43.9)	
High	139	28.4(24.5–32.5)	238	48.6(44.2–53)	113	23(19.5–27)		65	22.9(18.3–28.2)	127	44.7(39-50.6)	92	32.4(27.2–38.1)	
**Alcohol consumption-no (%)**							0.682							0.323
Yes	408	41.4(38.4–44.6)	406	41.3(38.2–44.4)	170	17.3(15-19.8)		0	0	5	55.6(19.5–86.6)	4	44.4(13.4–80.5)	
No	1466	40.5(38.9–42.1)	1551	42.8(41.2–44.4)	606	16.7(15.5–18)		984	18.6(17.6–19.7)	2101	39.7(38.4–41)	2207	41.7(40.4–43)	
**Cigarette smoking-no (%)**							< 0.001							< 0.001
Never	744	33.8(31.9–35.8)	1040	47.3(45.2–49.4)	415	18.9(17.3–20.6)		948	18.4(17.3–19.4)	2069	40(38.7–41.4)	2150	41.6(40.3–43)	
Current	830	51.9(49.5–54.4)	562	35.2(32.9–37.5)	206	12.9(11.3–14.6)		26	32.5(23-43.7)	26	32.5(23-43.7)	28	35(25.2–46.3)	
Former	300	37.1(33.8–40.4)	355	43.8(40.4–47.3)	155	19.1(16.6–22)		10	18.5(10.1–32)	11	20.4(11.4–33.6)	33	61.1(47.2–73.4)	
**Opium consumption- no (%)**							< 0.001							0.046
Yes	1040	48.7(46.6–50.9)	791	37.1(35-39.1)	303	14.2(12.8–15.7)		39	18.5(13.8–24.4)	68	32.2(26.2–38.9)	104	49.3(42.5–56.1)	
No	834	33.7(31.9–35.6)	1166	47.2(45.2–49.1)	473	19.1(17.6–20.7)		945	18.6(17.5–19.7)	2038	40(38.7–41.4)	2107	41.4(40-42.8)	

Note: Data are given as absolute number n (percentage). p Values for differences between categories were obtained using the χ2 for categorical variables

Abbreviations: BMI: body mass index; WSI: wealth score index

There were significant differences for variables age (male: P = 0.003 and female: P < 0.001), marital status (male: P = 0.019 and female: P = 0.022), educational level (both gender P < 0.001), physical activity (both gender P < 0.001), wealth status index (male: P < 0.001 and female: P = 0.001), smoking habit (both gender P < 0.001) and opium consumption (male: P < 0.001 and female: P = 0.046) between adults with overweight/obese compared to those with normal weight.

The prevalence of overweight and obesity was significantly lower in the male group aged ≥ 56 years old compared to younger adults (P = 0.003); Moreover, the prevalence of obesity was higher in female subjects aged > 45 (P < 0.001) (Table 1).

Table 2 represents the prevalence of abdominal obesity according to demographic, socio-economic and lifestyle factors. The prevalence of abdominal obesity in females was significantly higher in age group ≥ 56 years and education level ≤ 5 years (P < 0.001). Moreover, there were significant difference between the prevalence of abdominal obesity in the categorical levels of physical activity and WSI in both genders (P < 0.001). The highest prevalence of abdominal obesity in females was in age group ≥ 56 years and education level ≤ 5 years (P < 0.001). In male subjects, the highest prevalence of abdominal obesity was in high WSI group and former smokers (P = 0.001 and P < 0.001 respectively), while it was lower in opium users (P = 0.001). (Table 2).

**Table 2:** Prevalence of demographic, selected medical and laboratory characteristics of study participants by Abdominal obesity

Characteristics	Abdominal obesity in Male					Abdominal obesity in Female				
	Normal (n = 3819)	% (CI)	Abnormal (n = 832)	% (CI)	P-Value	Normal (n = 778)	% (CI)	Abnormal (n = 4548)	% (CI)	P-Value
**Age- yr. no (%)**					0.480					< 0.001
35–45	1429	82.2(80.3–83.9)	310	17.8(16.1–19.7)		443	22.3(20.6–24.2)	1540	77.7(75.8–79.4)	
46–55	1141	83(80.1-8/4.9)	234	17(15.1–19.1)		199	11.7(10.3–13.4)	1496	88.3(86.6–89.7)	
≥ 56	1249	81.3(79.2–83.1)	288	18.7(16.9–20.8)		136	8.2(7-9.7)	1512	91.8(90.3–93)	
**Marital status- no (%)**					0.669					0.379
Single	87	80.6(71.9–87.1)	21	19.4(12.9–28.1)		106	15.7(13.2–18.7)	568	84.3(81.3–86.8)	
Married	3733	82.2(81-83.2)	811	17.8(16.8–19)		672	14.5(13.5–15.5)	3980	85.5(84.5–86.5)	
**Education-no (%)**					0.995					< 0.001
≤ 5 years	941	82.2(79.9–84.3)	204	17.8(15.7–20.1)		257	11(9.8–12.3)	2089	89(87.7–90.2)	
6–12 years	2076	82.1(80.5–83.5)	454	17.9(16.5–19.5)		350	15.1(13.7–16.7)	1961	84.9(83.3–86.3)	
≥ 13 years	800	82.1(79.6–84.4)	174	17.9(15.6–20.4)		170	25.6(22.4–29)	495	74.4(71-77.6)	
**Physical activity-no (%)**					< 0.001					< 0.001
Low	1155	78.4(76.2–80.4)	319	21.6(19.6–23.8)		128	11.9(10.1–13.9)	950	88.1(86.1–89.9)	
Moderate	1372	82.4(80.5–84.2)	293	17.6(15.8–19.5)		460	14.1(12.9–15.3)	2808	85.9(84.7–87.1)	
Heavy	1293	85.5(83.6–87.2)	220	14.5(12.9–16.4)		190	19.4(17–22)	790	80.6(78–83)	
**WSI- no (%)**					0.001					< 0.001
Low	772	85.8(83.3–87.9)	128	14.2(12.1–16.7)		207	14.4(12.6–16.3)	1236	85.6(83.7–87.4)	
Low-middle	1082	82(79.9–84)	237	18(16-20.1)		193	12.5(11-14.3)	1349	87.5(85.7–89.1)	
Middle-high	1583	81.6(79.8–83.3)	357	18.4(16.7–20.2)		311	15.2(13.7–16.8)	1740	84.8(83.2–86.3)	
High	380	77.6(73.6–81)	110	22.4(19-26.4)		67	23.6(19–29)	217	76.4(71.1–81)	
**Alcohol consumption-no (%)**					0.768					0.214
Yes	805	81.8(79.3–84.1)	179	18.2(15.9–20.7)		0	0	9	100(NA)	
No	2977	82.2(80.9–83.4)	644	17.8(16.6–19.1)		774	14.6(13.7–15.6)	4518	85.4(84.4–86.3)	
**Cigarette smoking-no (%)**					< 0.001					0.534
Never	1760	80.1(78.3–81.7)	438	19.9(18.3–21.6)		757	14.7(13.7–15.6)	4410	85.3(84.4–86.3)	
Current	1377	86.2(84.4–87.8)	221	13.8(12.2–15.6)		12	15(8.6–24.8)	68	85(75.2–91.4)	
Former	645	79.7(76.8–82.3)	164	20.3(17.6–23.2)		5	9.3(3.8–20.9)	49	90.7(79.1–96.2)	
**Opium consumption- no (%)**					0.001					0.176
Yes	1796	84.2(82.5–85.7)	338	15.8(14.3–17.4)		24	11.4(7.7–16.5)	187	88.6(83.5–92.3)	
No	1986	80.4(78.8–81.9)	485	19.6(18.1–21.2)		750	14.7(13.8–15.7)	4340	85.3(84.3–86.2)	

Abbreviations: BMI: body mass index; WSI: wealth score index

Table 3 represents the association between related risk factors and overweight/obesity and abdominal obesity, using the univariate and multivariate analyses. The related risk factors associated with obesity in univariate analysis were also assessed and the subjects with overweight/obesity and abdominal obesity were compared with the normal subjects (Table 3). The odds of having overweight/obesity and abdominal obesity were estimated for nine factors: age, gender, marital status, education level, physical activity, WSI, alcohol consumption, cigarette smoking, and opium consumption (Table 3). In the univariate analysis, it was found that all of these factors were significantly associated with overweight/obesity except for age group ≥ 56 years and married and in the abdominal obesity except for low-middle group of WSI.

**Table 3:** Logistic regression analysis for the association of overweight/obesity and abdominal obesity with demographic, socio-economic and lifestyle factors

characteristics	Overweight/ Obesity OR (95% CI)		Abdominal obesity OR (95% CI)	
	Univariate model	Multivariate model	Univariate model	Multivariate model
**Age. year**				
35–45	1	1	1	1
46–55	1.24(1.11–1.38)	1.32(1.18–1.49)	1.31(1.19–1.44)	1.48(1.29–1.70)
≥ 56	1.09(0.98–1.21)	1.19(1.06–1.35)	1.32(1.20–1.45)	1.68(1.45–1.95)
**Gender**				
Male	1	1	1	1
Female	3.02(2.75–3.30)	2.20(1.95–2.48)	26.84(24.12–29.86)	23.62(20.52–27.19)
**Marital status**				
Single	1	1	1	1
Married	0.85(0.72–1.01)	1.19(0.99–1.42)	0.36(0.30–0.42)	1.13(0.91–1.40)
**Education**				
≤ 5 years	1	1	1	1
6–12 years	0.90(0.82–0.99)	1.02(0.91–1.14)	0.52(0.48–0.57)	0.87(0.76–0.99)
≥ 13 years	0.80(0.71–0.91)	0.73(0.62–0.86)	0.36(0.32–0.41)	0.57(0.47–0.69)
**Physical activity**				
Low	1	1	1	1
Moderate	1.24(1.11–1.38)	0.90(0.80–1.01)	1.71(1.55–1.89)	0.82(0.72–0.94)
Heavy	0.67(0.59–0.75)	0.66(0.58–0.75)	0.69(0.62–0.77)	0.60(0.51–0.70)
**WSI**				
Low	1	1	1	1
Low-middle	1.29(1.14–1.45)	1.37(1.21–1.56)	0.89(0.80-1.00)	1.21(1.04–1.42)
Middle-high	1.39(1.24–1.55)	1.52(1.34–1.73)	0.79(0.72–0.88)	1.23(1.05–1.44)
High	1.42(1.19–1.71)	1.91(1.54–2.35)	0.53(0.45–0.62)	1.38(1.08–1.76)
**Alcohol consumption**				
No	1	1	1	1
yes	0.54(0.47–0.62)	1.30(1.11–1.52)	0.17(0.14–0.20)	1.36(1.12–1.65)
**Cigarette smoking**				
Never	1	1	1	1
Current	0.29(0.26–0.32)	0.54(0.47–0.63)	0.11(0.09–0.12)	0.64(0.53–0.78)
Former	0.53(0.46–0.62)	0.95(0.80–1.13)	0.17(0.14–0.20)	0.95(0.77–1.16)
**Opium consumption**				
No	1	1	1	1
Yes	0.36(0.33–0.40)	0.67(0.59–0.76)	0.16(0.15–0.18)	0.80(0.68–0.95)

The adjusted model is adjusted for confounding variables age (continuous variable), gender (male, female), education years (categorical variable), wealth status index (categorical variable), and the variables related to lifestyle: cigarette smoking (categorical variable), alcohol consumption (yes, no), opium consumption (yes, no), physical activity level (categorical variable) and marital status (categorical variable)

After adjustment, the variables including age, gender, education ≥ 13 years, heavy physical activity, WSI, alcohol consumption, current smoking and opium consumption were shown to be significantly associated with overweight/obesity. On the other hand, all assessed risk factors except married and former smoking had significant relationship with abdominal obesity (Table 3).

In the crude regression model, the odds ratio (OR) of overweight/ obesity (OR: 3.02, 95% CI 2.75 to 3.30) and abdominal obesity (OR: 26.84, 95% CI 24.12 to 29.86) were higher among women compared with men, and after adjustment for confounders this association remained significant, although it slightly diminished. In the regression model, the odds of abdominal obesity for all categories of age (46–55 years old OR: 1.31, 95% CI 1.19 to 1.44 and age ≥ 56 OR: 1.32, 95% CI 1.20 to 1.45) and the odds of overweight/ obesity for all categories of age (46–55 years old OR: 1.24, 95% CI 1.11 to 1.38 and age ≥ 56 years old OR: 1.32, 95% CI 1.20 to 1.42) were higher compared to reference age group (35–45 years) and this association remained significant after adjustment for the confounders. In terms of age, people with age 46–55 had highest prevalence of overweight/ obesity than 35–45 years old individuals (OR: 1.32, 95% CI 1.18 to 1.49) and people with age ≥ 56 had highest prevalence of central obesity than 35–45 years old individuals (OR: 1.68, 95% CI 1.45 to 1.95).

In the regression model, the odds of overweight and obesity was lowest in education ≥ 13 years (OR: 0.80, 95% CI 0.71 to 0.91), in comparison to people with education ≤ 5 years and this association persisted after adjustment for confounders (OR: 0.73, 95% CI 0.62 to 0.86). Also, in adjusted model the odds of abdominal obesity in categories of education for education ≥ 13 was lowest in comparison with people with education ≤ 5 years (OR: 0.57, 95% CI 0.47 to 0.69). Results of multivariate logistic regression analysis showed that moderate and heavy physical activity decreased the ORs of overweight/obesity and abdominal obesity.

Regarding Socio-economic status (WSI), in adjusted model the odds of overweight/obesity and abdominal obesity increased significantly with the improvement of socio-economic status.

The ORs of overweight/obesity in the un-adjusted model were significantly lower for current cigarette smoking (OR: 0.29, 95% CI 0.26–0.32), and opium consumption (OR: 0.36, 95% CI 0.33–0.40), and remained significant in the adjusted models (OR: 0.54, 95% CI 0.47–0.63 and OR: 0.67: 0.67, 95% CI 0.59–0.76 respectively). Similarly, lower ORs of abdominal obesity were seen with current cigarette smoking and opium consumption in both the un-adjusted and adjusted models.

Alcohol consumption decreased the odds of overweight/obesity and central obesity in the univariate models (OR: 0.54, 95% CI 0.47–0.62 and OR: 0.17, 95% CI 0.14–0.20), while after adjusting for confounders, these odds were significantly increased (OR: 1.30, 95% CI 1.11–1.52 and OR: 1.36, 95% CI 1.12–1.65 respectively). In order to find which confounding factor influences this result, we did the stepwise adjustment. The result showed that after adding opium to the model, effect of alcohol became significant.

## Discussion

According to the results, the overall prevalence of overweight and obesity were 40.97% (39.70% in women and 42.42% in men) and 30.13% (41.73% in women and 16.85% in men), respectively. In the study by Tabrizi et al., that conducted in the northwestern of Iran [21] similar with this study, around 63.6% of the participants were either overweight or obese. In another study from southwest of Iran, the total prevalence of overweight and obesity were almost similar to the results of this study (38.7% and 26.5%)[22]. These rates may reflect a significant national obesity problem. In the current study higher rates of overweight and obesity were observed when compared with estimates for the Spanish (17%) [23], Turkish (19%) [24] and Pakistan (25.0%) [25] adult population.

The total prevalence rate of abdominal obesity was 53.92% (85.39% in women and 17.88% in men). This rate was lower than the results of the study by Tabrizi et al., [21] who reported 75.2% central obesity (81.4%in women and 68.6% in men). In the Persian Giulan cohort study [26], central obesity had the highest prevalence (75.8%), and this index was more than 98% in females. But, the prevalence of abdominal obesity was 32.1% (57.2% in women and 15.8% in men) in Golestan (north of Iran) [27] and 21.2% in Ahvaz (south of Iran) [28]. These differences can be related to the lifestyle of people in large cities compared to small cities, and environmental conditions such as humidity that causes more sweating. The prevalence of abdominal obesity was 24.1% in Egypt [29], 30.5% in Australia[30], and 64.4% in Oman[31].

In the regression model, the ORs of overweight/obesity and abdominal obesity were higher among women compared with men. This results are in agreement with other studies in Iran [21, 22, 32–34] and other countries [35]. However, these results were dissimilar to the studies in China [36] and Japan[37]. This difference can be due to the cultural and occupational differences of women in Iranian society, especially in the south of the country compared to other countries. Moreover, the prevalence of abdominal obesity in females was significantly higher in age group ≥ 56 years and this association remained significant for both of them after adjustment for the confounders. In the study by Tabrizi et al.,[21], 45% of women in the 46–65 year old age group were obese. In agreement with the results of this study, age was considered as a predictor of abdominal obesity in other studies [27, 38–40].

After adjustment, the variable education ≥ 13 years, was shown to be significantly associated with overweight/obesity. These results are in according with some studies [41, 42], but there are other studies in Iran which found no relation between education and obesity [27, 43].

The multivariate analysis showed that moderate and heavy physical activity decreased the risk of overweight/obesity and abdominal obesity. Low physical activity and central obesity were associated in other studies reported from Iran and other countries [27, 32, 44, 45].

Lower ORs of overweight/obesity and abdominal obesity were seen in current cigarette smoking. Consistent with our results, another study in the northwest of Iran found that nonsmokers were more likely to be overweight or obese and abdominally obese [21]. The present study showed that opium consumption decreased and alcohol consumption increased the odds of overweight/obesity and abdominal obesity. These results were in line with other studies [46–49].

According to the results of the present study around 71.1% of adult population in this region have overweight/ obesity and half of them have central obesity, which emphasizes the need for comprehensive preventive strategies to reduce energy consumption. There is an urgent need for public health prevention strategies to help modify health behaviors to decrease obesity and its subsequent complications such as diabetes, hypertension, and coronary artery disease. An increase in physical activity by walking, bicycle riding, and sports, lifestyle changes by replacing high fiber and low-fat diets for routine diets are recommended.

The strengths of this study are the population-based design and the sample size. None of the previous studies had a sample size as much as our samples. Moreover, another noteworthy point of this study is that it was conducted by the experts and trained interviewers and all data carefully recorded. The limitations of this study are as follows: first, in this study, data on habits was based on the self-reporting and the individual’s memory, raising the possibility of errors. Second, in the present study, other parameters such as dietary pattern, stress and environmental factors were not assessed. Therefore, this issue should be taken into account in the future studies.

## Conclusions

The results of this study added more evidence related to the overweight/obesity and abdominal obesity in this region. These findings may help public health professionals, to develop the strategies that prevent of obesity in the community.

## Electronic supplementary material

Below is the link to the electronic supplementary material.

Supplementary Material 1

## Data Availability

The datasets used during the current study are available on the Persian Adult Cohort Study Center, Rafsanjan University of Medical Sciences, Iran. The data is not available publicly. However, upon a reasonable request, the data can be obtained from the correspondence.
