# Supplementary material for: The prevalence and associated factors of overweight/obesity and abdominal obesity in South-eastern of Iran: a cross-sectional study based on Rafsanjan cohort study
Source: BMC Public Health. 2023 May 11;23:861. doi: 10.1186/s12889-023-15700-0 (PMC10176792; doi:10.1186/s12889-023-15700-0)
Supplement: Supplementary file 1 — Supplementary Material 1 [file 12889_2023_15700_MOESM1_ESM.doc]

| Table S1. Stepwise adjustment for the association of overweight/obesity and abdominal obesity with demographic, socio-economic, lifestyle factors. | | |
| --- | --- | --- |
| Abdominal obesity  OR (95% CI) | Overweight/ Obesity  OR (95% CI) | characteristics |
| Multivariate model | Multivariate model |
|  | **Step 1** | |
|  | **Alcohol consumption** | |
| 1 | 1 | No |
| 0.17(0.14-0.20) | 0.54(0.47-0.62) | yes |
|  |  | **Step 2** |
|  | **Alcohol consumption** | |
| 1 | 1 | No |
| 0.17(0.15-0.21) | 0.55(0.48-0.64) | yes |
|  |  | **Age. year** |
| 1 | 1 | 35-45 |
| 1.15(1.04-1.27) | 1.17(1.05-1.30) | 46-55 |
| 1.15(1.05-1.27) | 1.03(0.92-1.14) | ≥56 |
|  | **Step 3** | |
|  | **Alcohol consumption** | |
| 1 | 1 | No |
| 1.19(0.99-1.43) | 1.01(0.87-1.16) | yes |
|  |  | **Age. year** |
| 1 | 1 | 35-45 |
| 1.52(1.33-1.74) | 1.23(1.1-1.37) | 46-55 |
| 1.82(1.59-2.08) | 1.11(1-1.23) | ≥56 |
|  |  | **Gender** |
| 1 | 1 | Male |
| 29.2(25.97-32.84) | 3.01(2.7-3.31) | Female |
|  | **Step 4** | |
|  | **Alcohol consumption** | |
| 1 | 1 | No |
| 1.20(1-1.43) | 1.02(0.88-1.18) | yes |
|  |  | **Age. year** |
| 1 | 1 | 35-45 |
| 1.52(1.33-1.74) | 1.23(1.1-1.37) | 46-55 |
| 1.84(1.61-2.1) | 1.13(1.01-1.26) | ≥56 |
|  |  | **Gender** |
| 1 | 1 | Male |
| 29.72(26.35-33.53) | 3.11(2.82-3.43) | Female |
|  |  | **Marital status** |
| 1 | 1 | Single |
| 1.16(0.94-1.43) | 1.31(1.1-1.57) | Married |
|  | **Step 5** | |
|  | **Alcohol consumption** | |
| 1 | 1 | No |
| 1.02(1-1.45) | 1.03(0.89-1.19) | yes |
|  |  | **Age. year** |
| 1 | 1 | 35-45 |
| 1.52(1.33-1.74) | 1.23(1.10-1.38) | 46-55 |
| 1.85(1.61-2.11) | 1.17(1.05-1.31) | ≥56 |
|  |  | **Gender** |
| 1 | 1 | Male |
| 30.04(26.6-33.92) | 3.25(2.95-3.60) | Female |
|  |  | **Marital status** |
| 1 | 1 | Single |
| 1.12(0.9-1.38) | 1.17(0.97-1.40) | Married |
|  | **Wealth Status Index** | |
| 1 | 1 | Low |
| 1.20(1.03-1.4) | 1.42(1.25-1.60) | Low-middle |
| 1.15(1-1.33) | 1.59(1.41-1.79) | Middle-high |
| 1.17(0.93-1.46) | 1.93(1.60-2.34) | High |
|  |  | **Step 6** |
|  | **Alcohol consumption** | |
| 1 | 1 | No |
| 1.17(0.97-1.41) | 1.01(0.87-1.17) | yes |
|  |  | **Age. year** |
| 1 | 1 | 35-45 |
| 1.43(1.25-1.64) | 1.22(1.09-1.37) | 46-55 |
| 1.71(1.48-1.97) | 1.16(1.04-1.31) | ≥56 |
|  |  | **Gender** |
| 1 | 1 | Male |
| 29.12(25.76-32.93) | 3.24(2.92-3.58) | Female |
|  |  | **Marital status** |
| 1 | 1 | Single |
| 1.10(0.88-1.36) | 1.16(0.96-1.38) | Married |
|  | **Wealth Status Index** | |
| 1 | 1 | Low |
| 1.24(1.07-1.45) | 1.42(1.25-1.61) | Low-middle |
| 1.29(1.10-1.5) | 1.63(1.44-1.86) | Middle-high |
| 1.49(1.16-1.9) | 2.13(1.73-2.62) | High |
|  |  | **Education** |
| 1 | 1 | ≤5 years |
| 0.89(0.78-1.02) | 1.04(0.93-1.16) | 6-12 years |
| 0.63(0.52-0.76) | 0.85(0.73-1) | ≥ 13 years |
|  | **Step 7** | |
|  | **Alcohol consumption** | |
| 1 | 1 | No |
| 1.15(0.95-1.38) | 1.00(0.86-1.15) | yes |
|  |  | **Age. year** |
| 1 | 1 | 35-45 |
| 1.41(1.23-1.62) | 1.21(1.08-1.35) | 46-55 |
| 1.62(1.4-1.87) | 1.12(1.00-1.26) | ≥56 |
|  |  | **Gender** |
| 1 | 1 | Male |
| 28.65(25.25-32.50) | 3.09(2.78-3.43) | Female |
|  |  | **Marital status** |
| 1 | 1 | Single |
| 1.13(0.91-1.40) | 1.18(0.99-1.42) | Married |
|  | **Wealth Status Index** | |
| 1 | 1 | Low |
| 1.23(1.06-1.44) | 1.41(1.24-1.60) | Low-middle |
| 1.27(1.1-1.49) | 1.62(1.42-1.84) | Middle-high |
| 1.45(1.13-1.85) | 2.07(1.68-2.55) | High |
|  |  | **Education** |
| 1 | 1 | ≤5 years |
| 0.87(0.76-1) | 1.02(0.91-1.14) | 6-12 years |
| 0.60(0.49-0.72) | 0.81(0.69-0.95) | ≥ 13 years |
|  |  | **Physical activity** |
| 1 | 1 | Low |
| 0.84(0.74-0.97) | 0.94(0.84-1.05) | Moderate |
| 0.61(0.52-0.71) | 0.66(0.60-0.76) | Heavy |
|  | **Step 8** | |
|  | **Alcohol consumption** | |
| 1 | 1 | No |
| 1.28(1.06-1.56) | 1.20(1.03-1.40) | yes |
|  |  | **Age. year** |
| 1 | 1 | 35-45 |
| 1.45(1.26-1.67) | 1.28(1.14-1.44) | 46-55 |
| 1.67(1.44-1.93) | 1.18(1.05-1.33) | ≥56 |
|  |  | **Gender** |
| 1 | 1 | Male |
| 25.4(22.21-29.05) | 2.47(2.2-2.77) | Female |
|  |  | **Marital status** |
| 1 | 1 | Single |
| 1.14(0.91-1.41) | 1.20(1-1.44) | Married |
|  | **Wealth Status Index** | |
| 1 | 1 | Low |
| 1.23(1.05-1.43) | 1.40(1.23-1.59) | Low-middle |
| 1.25(1.07-1.46) | 1.56(1.37-1.77) | Middle-high |
| 1.39(1.09-1.78) | 1.92(1.56-2.37) | High |
|  |  | **Education** |
| 1 | 1 | ≤5 years |
| 0.86(0.75-0.99) | 1.01(0.90-1.13) | 6-12 years |
| 0.57(0.47-0.69) | 0.74(0.62-0.87) | ≥ 13 years |
|  |  | **Physical activity** |
| 1 | 1 | Low |
| 0.83(0.72-0.95) | 0.91(0.81-1.02) | Moderate |
| 0.6(0.52-0.7) | 0.66(0.58-0.75) | Heavy |
|  | **Opium consumption** | |
| 1 | 1 | No |
| 0.7(0.6-0.81) | 0.55(0.49-0.62) | Yes |
|  |  | **Step 9** |
|  | **Alcohol consumption** | |
| 1 | 1 | No |
| 1.36(1.12-1.65) | 1.30(1.11-1.52) | yes |
|  |  | **Age. year** |
| 1 | 1 | 35-45 |
| 1.48(1.29-1.70) | 1.32(1.18-1.49) | 46-55 |
| 1.68(1.45-1.95) | 1.19(1.06-1.35) | ≥56 |
|  |  | **Gender** |
| 1 | 1 | Male |
| 23.62(20.52-27.19) | 2.20(1.95-2.48) | Female |
|  |  | **Marital status** |
| 1 | 1 | Single |
| 1.13(0.91-1.4) | 1.19(1-1.42) | Married |
|  | **Wealth Status Index** | |
| 1 | 1 | Low |
| 1.21(1.04-1.42) | 1.37(1.21-1.56) | Low-middle |
| 1.23(1.05-1.44) | 1.52(1.34-1.73) | Middle-high |
| 1.38(1.08-1.77) | 1.91(1.54-2.35) | High |
|  |  | **Education** |
| 1 | 1 | ≤5 years |
| 0.87(0.76-1) | 1.02(0.91-1.14) | 6-12 years |
| 0.57(0.47-0.69) | 0.73(0.62-0.86) | ≥ 13 years |
|  |  | **Physical activity** |
| 1 | 1 | Low |
| 0.82(0.72-0.94) | 0.9(0.8-1.01) | Moderate |
| 0.6(0.51-0.7) | 0.66(0.58-0.75) | Heavy |
|  | **Opium consumption** | |
| 1 | 1 | No |
| 0.8(0.68-0.95) | 0.67(0.59-0.76) | Yes |
|  |  | **Cigarette smoking** |
| 1 | 1 | Never |
| 0.64(0.53-0.78) | 0.54(0.47-0.63) | Current |
| 0.95(0.77-1.16) | 0.95(0.80-1.13) | Former |
